# Nutrient balancing of the adult worker bumblebee (*Bombus terrestris*) depends on the dietary source of essential amino acids

**DOI:** 10.1242/jeb.114249

**Published:** 2015-03

**Authors:** Daniel Stabler, Pier P. Paoli, Susan W. Nicolson, Geraldine A. Wright

**Affiliations:** 1 Centre for Behaviour and Evolution, Institute of Neuroscience, Newcastle University, Newcastle upon Tyne NE1 7RU, UK; 2 Department of Zoology and Entomology, University of Pretoria, Private Bag X20, Hatfield 0028, South Africa

**Keywords:** Carbohydrate, Protein, Geometric framework, *Apis*, Bee, Forager

## Abstract

Animals carefully regulate the amount of protein that they consume. The quantity of individual essential amino acids (EAAs) obtained from dietary protein depends on the protein source, but how the proportion of EAAs in the diet affects nutrient balancing has rarely been studied. Recent research using the Geometric Framework for Nutrition has revealed that forager honeybees who receive much of their dietary EAAs from floral nectar and not from solid protein have relatively low requirements for dietary EAAs. Here, we examined the nutritional requirements for protein and carbohydrates of foragers of the buff-tailed bumblebee *Bombus terrestris.* By using protein (sodium caseinate) or an equimolar mixture of the 10 EAAs, we found that the intake target (nutritional optimum) of adult workers depended on the source and proportion of dietary EAAs. When bees consumed caseinate-containing diets in a range of ratios between 1:250 and 1:25 (protein to carbohydrate), they achieved an intake target (IT) of 1:149 (w/w). In contrast to those fed protein, bees fed the EAA diets had an IT more biased towards carbohydrates (1:560 w/w) but also had a greater risk of death than those fed caseinate. We also tested how the dietary source of EAAs affected free AAs in bee haemolymph. Bees fed diets near their IT had similar haemolymph AA profiles, whereas bees fed diets high in caseinate had elevated levels of leucine, threonine, valine and alanine in the haemolymph. We found that like honeybees, bumblebee workers prioritize carbohydrate intake and have a relatively low requirement for protein. The dietary source of EAAs influenced both the ratio of protein/EAA to carbohydrate and the overall amount of carbohydrate eaten. Our data support the idea that EAAs and carbohydrates in haemolymph are important determinants of nutritional state in insects.

## Abstract (precis)

**Summary:** The essential amino acid profile of the bumblebee diet influences regulation of the amount of both protein and carbohydrate consumed.

## INTRODUCTION

Animals obtain essential amino acids (EAAs) by the consumption of plant or animal proteins. Proteins are digested into amino acid (AA) units, which are absorbed and then used to produce new proteins, generate ATP, make other amino acids or used as signals between cells. Because the need for AAs continues throughout an animal's lifespan, protein intake is actively regulated around a nutritional optimum that is determined by age, physiological state and reproductive capacity (Simpson and Raubenheimer, 2012). Animals regulate their protein intake by altering quantities of food eaten (Simpson et al., 2004) or by consuming a mixture of foods with the correct balance of protein and other macronutrients (Raubenheimer and Simpson, 1993; Simpson and Raubenheimer, 1993, 2012; Simpson et al., 2004). How the regulation of protein intake is accomplished by the body's ability to detect the need for essential amino acids (EAAs) is largely unknown (Morrison et al., 2012).

The protein source determines the proportion and types of AAs produced by its digestion (Boisen et al., 2000) and can affect macronutrient balancing (Lee, 2007; Altaye et al., 2010). The amount of protein consumed in the diet directly affects the concentration of free AAs in the blood/haemolymph (Zanotto et al., 1996; Abisgold and Simpson, 1988). For this reason, several authors have hypothesized that blood/haemolymph levels of AAs are a potential means by which the body detects AA nutritional sufficiency (Sanahuja and Harper, 1963; Peters and Harper, 1985; Simpson and Raubenheimer, 1993; Morrison et al., 2012; Solon-Biet et al., 2014). For example, haemolymph AA titre can directly influence feeding behaviour, as seen when injection with AA solutions reduces meal size and increases the time between meals in locusts (Abisgold and Simpson, 1988). Haemolymph EAA composition can also modulate gustatory sensitivity to AAs in taste neurons (Simpson and Simpson, 1992) and could interact with feeding circuits in the brain to regulate protein feeding. In mammals, neurons in the hypothalamus, which govern food intake and are sensitive to carbohydrate levels in the blood, also respond to specific AAs, including leucine (Karnani et al., 2011), and direct injection with AAs can reduce meal size (Jordi et al., 2013). However, few AAs have been identified that interact with these neurons and additional brain structures could also be involved (Schwartz, 2013).

The Geometric Framework for Nutrition is a modelling method that works on the principle that all animals need specific proportions of macronutrients for optimal performance (Simpson and Raubenheimer, 2012). This optimum, called the ‘intake target’, can be determined experimentally for a species with a given set of traits (sex, age, reproductive status) (Simpson and Raubenheimer, 1993; Raubenheimer and Simpson, 1997; Simpson et al., 2004). This is accomplished by either confining individuals to diets composed of specific proportions of macronutrients or by giving animals a choice of two diets with different macronutrient ratios and measuring the amount of food they consume as well as other performance indicators including lifespan, digestion efficiency, weight and health (Raubenheimer and Simpson, 1993; Simpson and Raubenheimer, 2012).

Adult workers of eusocial insects such as honeybees and ants are unusual because their requirements for dietary protein are very low (Pirk et al., 2010; Altaye et al., 2010; Paoli et al., 2014a,b; Dussutour and Simpson, 2009). For example, a recent study using the Geometric Framework estimated that foraging worker honeybees need 250 times less dietary EAAs than bee larvae (Paoli et al., 2014a,b); broodless honeybee ‘nurses’, in contrast, required five times more dietary EAAs than foragers (Paoli et al., 2014a). However, few other social insect species have been studied using the Geometric Framework. The buff-tailed bumblebee *Bombus terrestris* is a generalist pollinator that lives in eusocial colonies of a few hundred individuals. It is an important wild pollinator but has recently been domesticated and is now used extensively in commercial pollination systems. In comparison to honeybees, its biology is more similar to other wild bee species in that it does not store much food; instead pollen brought back to the colony is consumed quickly by colony residents and fed to brood, and only nectar is stored. In comparison to other wild pollinators, commercially reared colonies make it easy to study this species under lab conditions. At present, we know very little about the dietary requirements of bee species other than honeybees, and whether the low requirement for dietary protein is common to other species of eusocial insect workers. Furthermore, because workers mainly require protein for somatic maintenance, bees could be ideal models to test how the dietary intake of EAAs is regulated in the absence of sexual reproduction. Foraging worker bees are unusual because they derive a portion of their dietary EAAs from free AAs found in floral nectar (Gardener and Gillman, 2002; Petanidou et al., 2006; Nicolson and Thornburg, 2007) and readily consume solutions containing AAs.

Here, we use the Geometric Framework to identify the nutritional optimum for dietary EAAs and carbohydrates of the adult worker bumblebee (*B. terrestris*). Previous studies of its nutrition have shown that microcolonies compensate for protein levels in pollen: bumblebees eat relatively more pollen when its protein content is low (measured as %N) (Tasei and Aupinel, 2008). At present, however, very little is known about the macronutrient requirements of this bee species, in spite of the fact that it is widely used in commercial pollination and is an important model in laboratory studies. In these experiments, the IT for carbohydrates and a dietary source of EAAs (either the 10 EAAs or a protein, caseinate), was determined using an experimental design where bees were allowed to choose between a diet containing sucrose and a source of EAAs and sucrose alone. Using this design, we were also able to test whether the dietary source of EAAs influenced the IT. To gain insight into the mechanisms of nutrient regulation (Simpson and Raubenheimer, 1993), we also measured how the amount of AAs present in bumblebee haemolymph depended on the dietary protein source and concentration.

## RESULTS

### Nutrient balancing depends on the source of EAAs

**Fig. 1.**
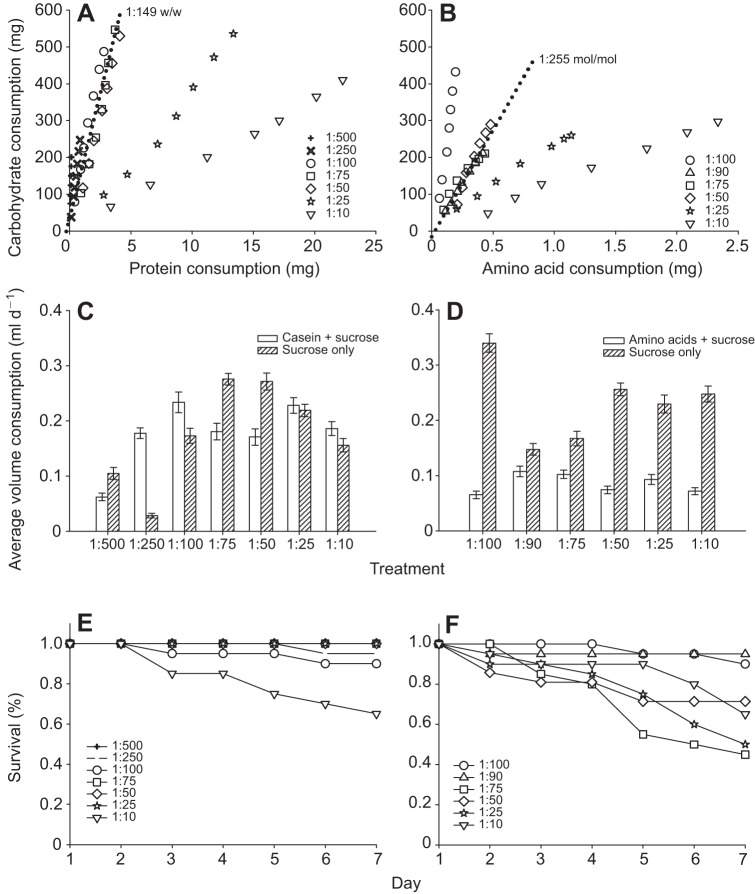
**Nutrient balancing towards an intake target depends on the dietary source of EAAs.** (A) Bees fed a choice of diets containing caseinate and 0.5 mol l^−1^ sucrose and 0.5 mol l^−1^ sucrose alone balanced their intake of protein and carbohydrate to an intake target of 1:149 w/w (P:C). (B) Bees fed with diets containing free EAAs and 0.5 mol l^−1^ sucrose and 0.5 mol l^−1^ sucrose alone achieved an intake target of 1:255 mol/mol (1:560 w/w). The dotted line in both panels illustrates the putative intake target. (C) The proportion of caseinate-sucrose diet to sucrose-only diet depended on the diet pair. (D) Bees fed the EAA-sucrose to sucrose-only diet consistently ate more of the sucrose-only diet than the diets containing free EAAs. (E) Bees fed with diets high in caseinate had a lower risk of mortality than bees fed diets high in free EAA (F). The data are for the same individuals in all panels. Error bars indicate s.e.m. *N*=20 bees per diet pair per panel.

The intake target of worker bumblebees depended on the dietary source of EAAs (Fig. 1A, B). Three of the diet solutions in each of the treatments (protein or EAAs) allowed bees to achieve their intake target. Bees fed with caseinate achieved an intake target of 1:149 (w/w) when given the option to eat from tubes containing 0.5 mol l^−1^ sucrose paired with the 1:100, 1:75 and 1:50 (w/w) caseinate and sucrose diets (Fig. 1A). Bees fed with both 0.5 mol l^−1^ sucrose and sucrose containing free EAA achieved an intake target of ∼1:255 (mol/mol) when fed with the 1:90, 1:75 and 1:50 (mol/mol) diets (Fig. 1B), which translates into an intake target of 1:560 w/w. Bees fed with the caseinate diets consumed approximately twice as much carbohydrate as those fed with the free EAA solutions when they were feeding on diets within the range over which they could achieve their intake target (1:50, 1:75, 1:100 w/w).

We calculated the intake target by measuring the total amount of food consumed by adult worker bumblebees over the course of the 7 day experiment. The proportion of protein to carbohydrate (P:C) or EAA:C in the diet solution had a strong effect on the amount of food eaten (Fig. 1). Bees fed with sucrose paired with sucrose-caseinate solutions (Fig. 1A) ate significantly more caseinate when they were given the 1:25 and 1:10 (w/w) diets than the bees fed with any of the other diets (Table 1, Šidák's *post hoc*, *P*<0.05). These diets had a much higher concentration of protein than the bees' intake target. In contrast, bees fed with the diet pairs in which it was not possible for them to eat enough to achieve their intake target for protein (the 1:500 and 1:250 diets) ate less on average (Fig. 1A) but also ate significantly less carbohydrate than bees on the other caseinate diets (Table 1, Šidák's *post hoc*, *P*<0.05). Like the bees fed diets containing a high proportion of caseinate, bees fed the diets made of free EAAs ate significantly more EAAs when fed the diets with high EAA:C proportions (1:10 and 1:25 mol/mol) (Fig. 1B, Table 1, Šidák's *post hoc*, *P*<0.05). These data show that bees have a set mean requirement for daily carbohydrate (supplementary material Fig. S1; ∼45 mg day^−1^) and prioritize their intake of carbohydrate over their intake of protein. Unlike the bees fed the dilute caseinate diets, however, bees fed the most dilute EAA:C diet (1:100) ate significantly more carbohydrate than those fed with the other diets (Table 1, Šidák's *post hoc*, *P*<0.05).

**Table 1.**
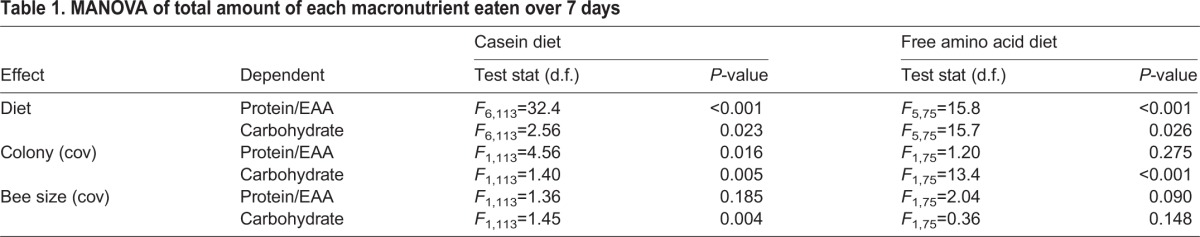
**MANOVA of total amount of each macronutrient eaten over 7 days**

In our analysis, we controlled for the colony of origin of the bees and found that it affected the amount of carbohydrate consumed in both sets of experiments (Table 1). The colony also influenced the amount of caseinate but not the amount of EAAs eaten (Table 1). In addition, we controlled for bee size and found that it influenced the amount of carbohydrate consumed when bees were fed the caseinate diets (Table 1). For both diet treatments, larger bees ate more carbohydrate (Pearson's correlation coefficient: caseinate, *r*=0.496, *P*<0.001; EAA, *r*=0.221, *P*=0.043).

The source of EAAs in diet also influenced the mean daily volume of each diet solution consumed by bees (Fig. 1C,D). Bees fed with the caseinate diets altered their intake to consume more sucrose-only solution when it was paired with a high-protein solution (e.g. 1:50 diet) but ate less of the sucrose-only solution when it was paired with a dilute protein source (e.g. 1:250) (Fig. 1C, two-way ANOVA, treatment×solution, *F*_6,234_=7.99, *P*<0.001). Bees fed with caseinate diets on the extreme ends of the range we tested (1:500, 1:25, 1:10) did not compensate in this way. Furthermore, larger bees ate a greater volume of the diet solutions on average [two-way ANOVA, weight (cov), *F*_1,249_=5.85, *P*=0.016]. The amount of food eaten also varied as a function of colony [two-way ANOVA, colony (cov), *F*_1,249_=9.07, *P*=0.003]. In contrast, bees fed with diets containing the 10 EAAs in 0.5 mol l^−1^ sucrose solution always ate more of the sucrose solution (Fig. 1D), and the amount of the EAA diet solution they consumed depended on the proportion of EAA:C (two-way ANOVA, trt×solution, *F*_5,223_=7.07, *P*<0.001). There was no effect of bee size on this relationship (two-way ANOVA, weight covariate, *F*_1,224_=1.05, *P*=0.306). The volume eaten varied as a function of colony [two-way ANOVA, weight (cov), *F*_1,249_=6.49, *P*=0.012].

### High concentrations of EAA in food increase the risk of mortality

Bees fed diets composed of caseinate had very low rates of mortality and their survival was largely unaffected by caseinate concentration in the diet (Coxreg, χ_1_^2^=2.79, *P*=0.095) (Fig 1E). However, the bees fed the highest concentration of caseinate (1:10) had a 3.9 times greater risk of dying than those fed the most dilute diet (1:500). Bees fed diets high in EAA, however, were more likely to die during the course of the experiment than those fed with caseinate (Fig. 1F, Coxreg, χ_1_^2^=5.78, *P*=0.016). The risk of dying increased as a function of the amount of EAAs in the diet; bees fed diets with EAA:C ratios less than 1:90 (mol/mol) had a 3–7 times greater risk of dying than those fed diets higher in carbohydrates (e.g. >1:90).

### Dietary source of EAAs influences the amount and proportion of sugars and AAs in haemolymph

**Fig. 2.**
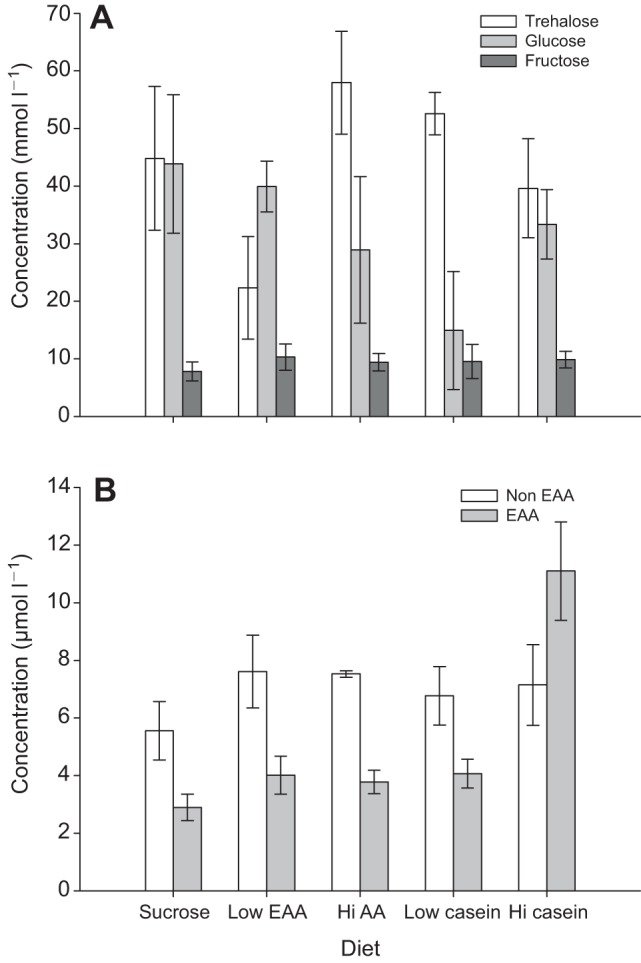
**Haemolymph sugars and amino acids depended on the concentration of protein or EAAs in diet.** (A) The sugars, glucose and trehalose, varied according to diet, but fructose did not. (B) Bees fed diets high in caseinate had almost twice the total average amount of EAAs in haemolymph as bees fed sucrose alone or any of the other diets. The mean amount of non-EAAs did not vary as a function of diet. Error bars indicate s.e.m. *N*_suc_=9, *N*_lowAA_=4, *N*_hiAA_=10, *N*_lowcas_=8, *N*_hicas_=6.

We confined bees to a specific diet and measured how diet influenced haemolymph nutrient composition. The ratio of P:C or EAA:C in the diet influenced the amount and proportion of sugars and amino acids in the bee haemolymph (Fig. 2). The main sugars we found in bumblebee haemolymph were trehalose, glucose and fructose (Fig. 2A); sucrose was also present, but at concentrations ≥2 orders of magnitude lower than the other sugars (0.380 mmol l^−1^; data not shown). The amount and proportion of sugars in bee haemolymph depended on the diet (GEE, treatment×sugar, χ_12_^2^=58.1, *P*<0.001). Of all the sugars we measured, trehalose was present in the haemolymph in the highest concentration, except in bees fed the low EAA diet (1:600 mol/mol). In these bees, glucose was at a higher concentration than trehalose (Šidák's *post hoc*, *P*<0.05). In all of the bees we sampled, fructose was present at an average concentration of ∼9.2±0.8 mmol l^−1^; in contrast to trehalose and glucose, fructose concentration did not vary as a function of the diet treatment (Fig. 2A, Šidák's *post hoc*, all *P*>0.05).

Haemolymph amino acid concentrations were also influenced by diet (Fig. 2B). The proportion of total EAAs to non-EAAs depended on diet (GEE, diet×AA class, χ_4_^2^=24.4, *P*<0.001). In all the diet treatments except the high caseinate diet (1:20 w/w), the bees had lower concentrations of EAAs than non-EAAs in haemolymph (Fig. 2B). The bees fed the high caseinate diet had almost three times the level of haemolymph EAAs compared with bees fed the other diets; in fact, six of the 10 EAAs (leucine, isoleucine, valine, methionine, threonine and lysine) were elevated in haemolymph when bees were fed this diet (Table 2). Total haemolymph EAAs were not significantly different for any of the other diet treatments (Šidák's *post hoc*, *P*>0.05). Interestingly, with the exception of bees fed sucrose, the mean concentration of non-EAAs was not strongly affected by diet treatment (Fig. 2B, Šidák's *post hoc*, *P*>0.05); the sucrose-only fed bees had significantly lower non-EAAs than those fed with the high EAA diet (Šidák's *post hoc*, *P*=0.042).

**Table 2.**
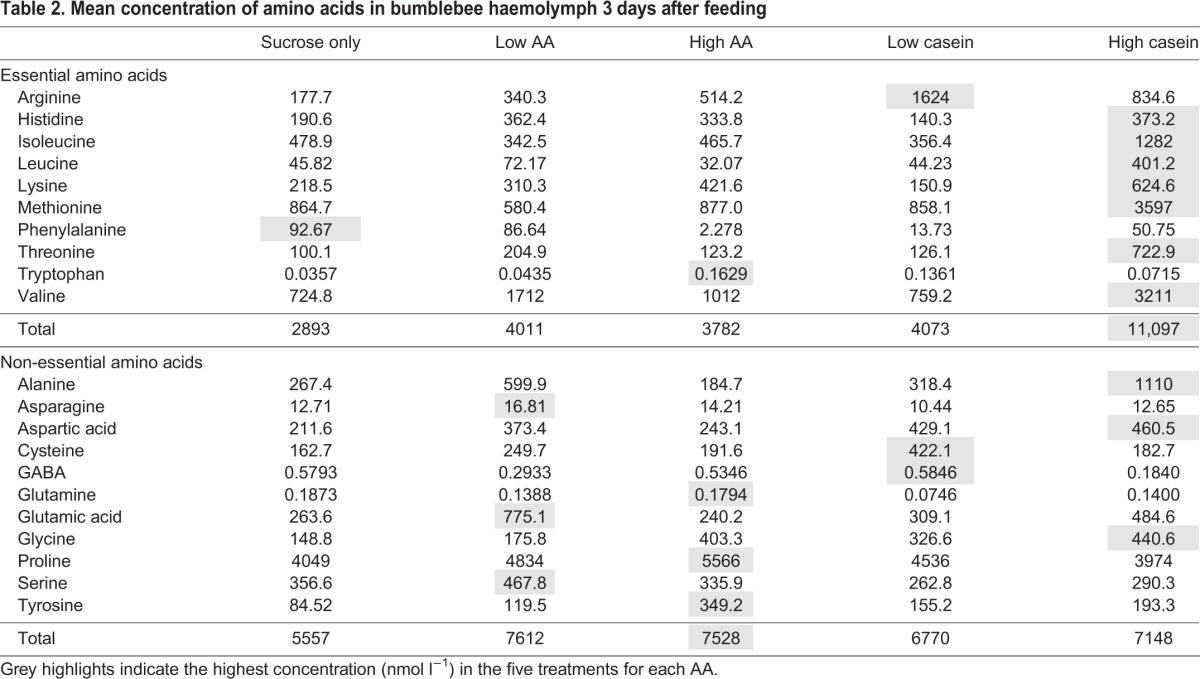
**Mean concentration of amino acids in bumblebee haemolymph 3 days after feeding**

To identify whether specific amino acids signalled protein/EAA sufficiency, it was necessary to identify whether diet influenced the amino acid profile of haemolymph. To do this, we tested whether the proportion of specific EAAs and non-EAAs in bumblebee haemolymph could predict the diet the bees were fed using canonical discriminant analysis (CDA). A CDA for the EAAs revealed that diet influenced the specific profile of AAs in bee haemolymph (Table 3). The first canonical discriminant function (function 1) separated the bees fed diets high in protein (high caseinate diet, 1:20 w/w) and the bees fed diets high in EAAs (high AA diet, 1:30 mol/mol) from all other groups (canonical discriminant function coefficients, Table 3). The main haemolymph AAs used to separate the bees fed the high caseinate diet from the other groups were leucine, threonine and valine (pooled within-group correlations, Table 3). In fact, these bees had ∼10× as much leucine in their blood as the bees fed the sucrose-only or low caseinate diets. Bees fed the high AA diet, however, had the lowest concentration of leucine of all the diets. The second discriminant function distinguished the bees fed sucrose only and the low AA diet from those fed with the low caseinate diet; the bees fed the low caseinate diet had relatively elevated levels of tryptophan and low levels of phenylalanine (Tables 2 and 3). The third and fourth discriminant functions did not significantly distinguish the groups.

**Table 3.**
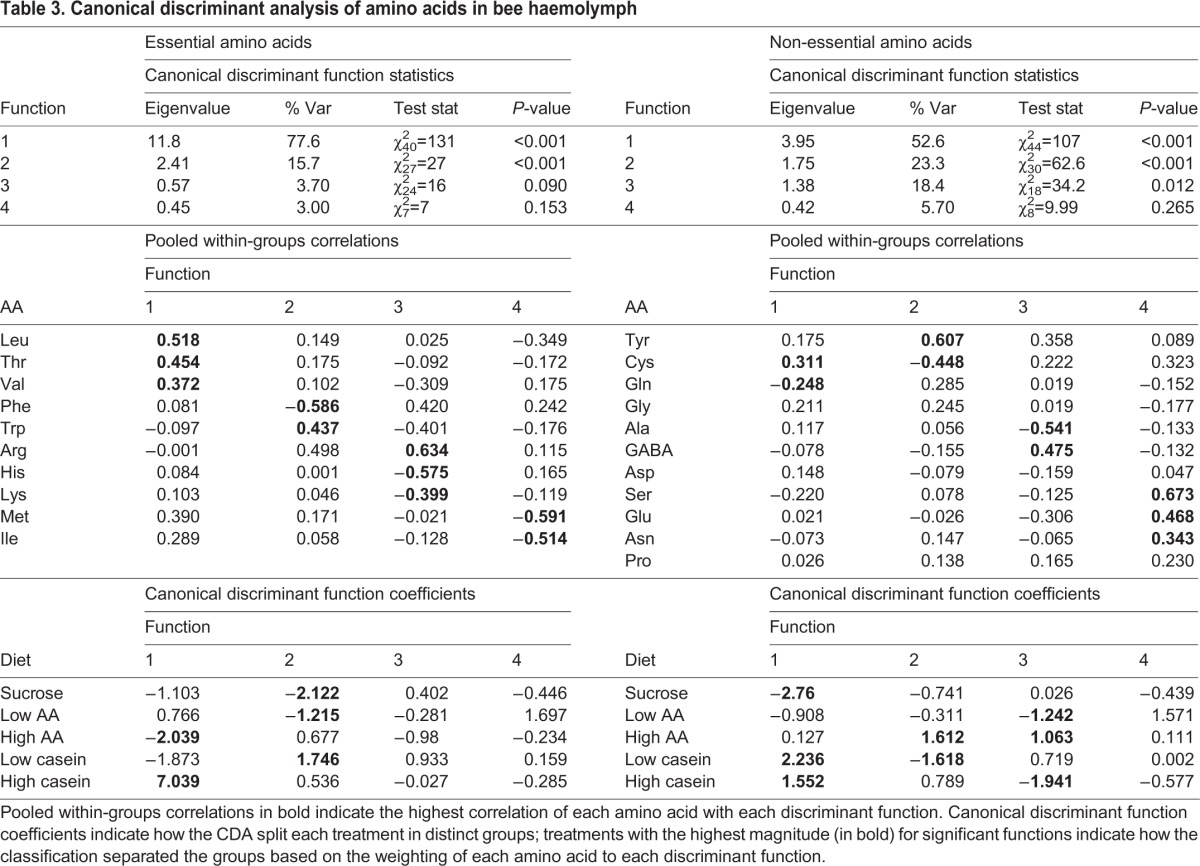
**Canonical discriminant analysis of amino acids in bee haemolymph**

A second CDA was performed for the non-essential AAs (Table 3). Three significant functions were produced. The first function distinguished the bees fed the sucrose-only diet from those fed caseinate based on the quantities of cysteine and glutamine (Tables 2 and 3). Cysteine was highest in concentration in the low caseinate diet and glutamine was highest in concentration in the sucrose-only diet. The second discriminant function distinguished the bees fed the low caseinate diets and those fed the high AA diets from the others based on the concentration of cysteine and tyrosine (Tables 2 and 3). The third distinguished the low AA diet and the high caseinate diet from the high AA diet; the low AA diet and the high caseinate diet had relatively greater concentrations of alanine and lower concentrations of GABA (Tables 2 and 3).

## DISCUSSION

Our experiments show that, like honeybees (Paoli et al., 2014a,b), bumblebee workers prioritize their intake of carbohydrates over the ingestion of dietary EAAs. The source of dietary EAAs influenced nutrient balancing: when bees were fed with a protein (caseinate), they ate a relatively higher proportion of P:C (intake target, 1:149 w/w) than bees fed with the equimolar, free EAA solutions (intake target, 1:255 mol/mol or 1:566 w/w). Interestingly, bees fed with caseinate also consumed almost twice as much carbohydrate as those fed with the free EAA solutions, even though proportionally their diets were skewed towards protein. The bees fed solutions of free EAAs required less of the EAA solution, consumed less carbohydrate and regulated their intake of the solution over a wider range of concentrations. Like other social insect workers studied previously (Dussutour and Simpson, 2009; Pirk et al., 2010; Paoli et al., 2014a,b), diets high in EAAs caused higher rates of mortality in adult worker bumblebees.

### Potential mechanisms for adjustment of protein/EAA intake

One of the most striking results of our study was that the amount and proportion of EAAs in food affected the regulation of EAA intake by individual bees. Bees fed the caseinate diet consumed ∼4× more of the sucrose-caseinate diet to meet their needs for EAAs than bees fed with diets containing equimolar concentrations of free EAAs. This could be as a result of incomplete digestion of the casein by the bees, resulting in a greater demand for the substrate, but we were unable to test the frass of the bees to confirm this. Furthermore, the costs of production of enzymes to digest casein might also cause a greater demand for EAAs. The bees fed with diets dilute in caseinate also exhibited difficulty in regulating their intake to compensate for the low amount of protein, suggesting that they might not be capable of post-ingestively detecting protein in the diet when it is present at concentrations less than 1:250 w/w.

The main difference between the caseinate-sucrose diet and the free EAA-sucrose diets was the proportion of EAAs (Table 4). The fact that the bees had to eat ∼4× more caseinate implies that some of the less abundant EAAs produced by the digestion of caseinate were important for the regulation of protein intake. With the exception of isoleucine and phenylalanine, the free EAA diet had greater proportions of all the other EAAs than caseinate. Two of these, threonine and valine, were ∼4× less concentrated in the caseinate diet (Table 4); the close match of their relative concentration to the factor by which the bumblebees ate more caseinate could imply that these two are particularly important for the regulation of EAA intake. Only lysine was less concentrated than threonine and valine; it was ∼13× less concentrated in the caseinate diet. Furthermore, phenylalanine and isoleucine were more concentrated than any of the other EAAs, perhaps indicating that they are less important in protein regulation. Our caseinate digest data also show that hydrolysis of caseinate yielded non-EAAs, which were not present in our equimolar, free EAA diet. The fact that bees had to ingest more caseinate to meet their needs for dietary EAAs – in spite of the fact that the caseinate diet also provided non-EAAs – indicates that non-EAAs play only a minor role in the regulation of food intake.

**Table 4.**
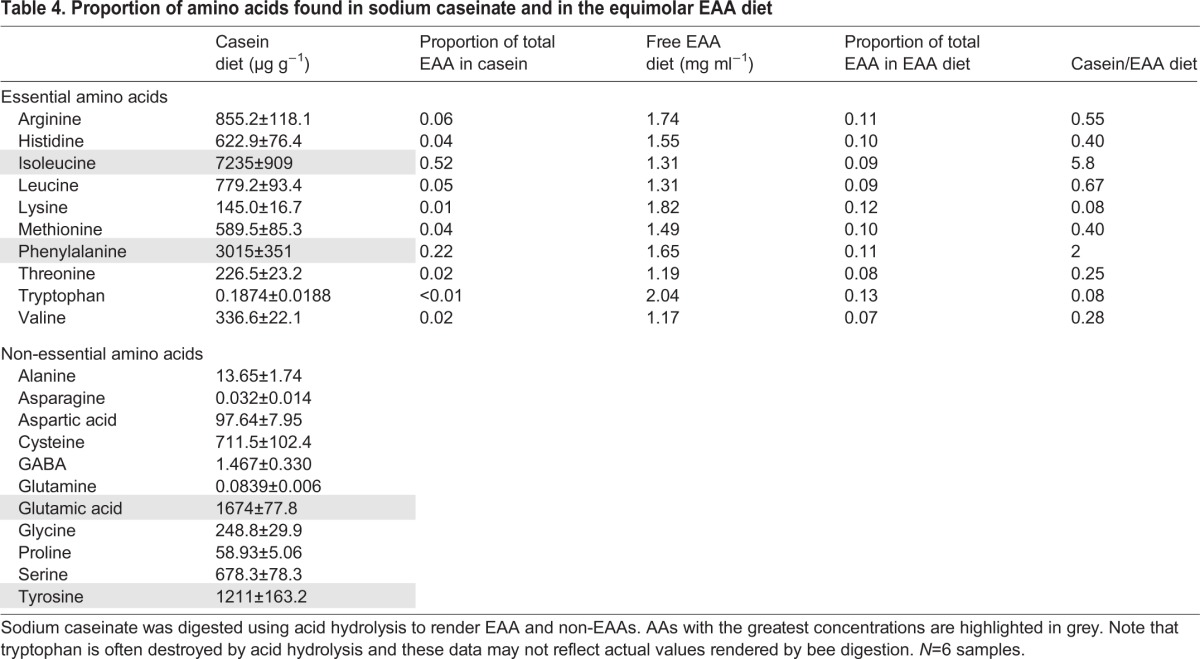
**Proportion of amino acids found in sodium caseinate and in the equimolar EAA diet**

The mechanisms that give rise to the regulation of protein intake are largely unknown (see Morrison et al., 2012 for a review). Dietary protein can affect signalling by peptides such as insulin (Buch et al., 2008), and this could indirectly provide a way of determining that protein has been eaten. A few studies have identified that an ‘over-abundance’ of specific amino acids can limit feeding behaviour (Purpera et al., 2012; Jordi et al., 2013). Leucine, for example, and the other branched-chain EAAs, isoleucine and valine, which activate the cellular target TOR (target of rapamycin), suppress feeding when they are present in abundance in diets fed to vertebrates (Peters and Harper, 1985) or injected directly into the brain centres involved in feeding, such as the hypothalamus (Blouet and Schwartz, 2010; Karnani et al., 2011; Laeger et al., 2014). In our experiments, only valine matched the predictions that branched-chain EAAs are important for protein regulation because it was four times less concentrated in the caseinate-sucrose diet than in the free EAA-sucrose diet; leucine was ∼30% less concentrated than in the free EAA diet and isoleucine was six times more concentrated (Table 4). These data imply either that valine is more important in protein regulation in insects or more than one EAA is necessary for the body to determine protein sufficiency. At present, there are few data to support the idea that the abundance of a single amino acid (e.g. valine or threonine) is used as a signal of protein sufficiency (Laeger et al., 2014). The more likely explanation is that more than one EAA is necessary for regulation of protein/EAA intake. Previous studies in locusts and rats have shown that the ingestion of several amino acids simultaneously often has a stronger effect on feeding than individual amino acids (Simpson et al., 1990; Karnani et al., 2011). It is possible that the ratios of the branched-chain amino acids to each other – or to combinations of other EAAs – are what affect the body's signals for protein sufficiency. Future studies that test how much each of the branched-chain AAs contributes to the intake of protein when they are present in a mixture of other EAAs will be necessary to identify whether all EAAs must be present in specific proportions to signal protein sufficiency.

### Dietary source of EAAs influences AAs in haemolymph

Our study is the first to establish that the dietary source of EAAs has a direct influence on haemolymph levels of AAs, especially EAAs, in an insect. In this experiment, we confined bees to diets that we predicted were either near the intake target or strongly unbalanced towards protein/EAA, with the expectation that we would be able to identify differences in the unbalanced diets that predicted the signal for protein sufficiency. As might be expected, our data show that over-ingestion of caseinate (arising from the need of the bees confined to this diet to ingest sufficient carbohydrate) resulted in ∼3–4× greater concentrations of 7 of the 10 EAAs in haemolymph. Notably, two of the branched-chain EAAs (leucine and valine) were elevated in haemolymph in spite of the fact that they were not the most abundant AAs present in the caseinate digest. A recent study also showed that rats chronically fed diets high in protein had elevated levels of the branched-chain AAs (leucine, isoleucine and valine) in their blood (Solon-Biet et al., 2014). Furthermore, in this same study, all other plasma AAs were negatively correlated with protein intake or not correlated at all. The selective elevation of the branched-chain EAAs in haemolymph when diets are high in protein could indicate that all other AAs from protein: (1) do not pass across the gut wall as readily; (2) are used more quickly by corporeal cells; or (3) are selectively excreted when they are in excess in haemolymph.

An interesting aspect of our study was that bumblebees did not accurately regulate their intake of EAAs when diets were high in protein or EAAs, as they over-ate EAAs in these diets, in spite of the fact that they were also given access to a sucrose-only diet. This suggests several possible explanations. The first is that bees cannot easily taste differences in the concentration of protein or EAAs in the diet, such that they passively over-ingest protein/EAAs in sugar solution. Few studies have examined the ability of bees to taste amino acids (Inouye and Waller, 1984; Roubik et al., 1995; Carter et al., 2006; Simcock et al., 2014; Hendriksma et al., 2014) and none have reported whether *B. terrestris* or other bees have the appropriate gustatory receptor neurons to detect them. The second is that post-ingestive mechanisms for the regulation of protein/EAA intake may be tuned to a specific range of concentrations of these amino acids, and if the concentration of protein/EAA is too high, the bees cannot adjust by reducing their intake.

### Bees prioritize carbohydrates over protein intake

We also observed that carbohydrate regulation depended on the dietary source of EAAs and the amount of protein eaten. Bees fed sucrose could only clearly regulate their intake around a specific daily quantity of carbohydrates (∼45±4 mg sucrose day^−1^, supplementary material Fig. S1). Bees fed with the free EAA-sucrose diets also regulated their intake of carbohydrates to ∼47±2 mg sucrose day^−1^; when caseinate was very dilute in the diet, as in the 1:250 and 1:500 caseinate-sucrose diets, bees regulated their intake to a similar amount (36±3 mg sucrose day^−1^). However, when caseinate was present at concentrations greater than the 1:250 diet, the bees not only consumed proportionally more caseinate (∼5±0.1 mg day^−1^), they also increased their total intake of carbohydrates to twice that of the bees on all the other diets (75±2 mg sucrose day^−1^). Our data could indicate that the brain integrates information about nutritional state using carbohydrates and EAAs simultaneously (Simpson and Raubenheimer, 2012), and that this calculation is done independently from an evaluation of the sufficiency of individual EAAs in the diet. Like other proteins, caseinate does not provide the same proportions of all EAAs (Table 4); to obtain sufficient specific EAAs, the bumblebees probably had to consume more caseinate (w/w) than the free EAA solution (w/w). However, they did this whilst also maintaining their intake target for a specific proportion of P:C and this forced them to eat, in total, more carbohydrates than the bees fed with the free EAA diets. Thus, our data show that the proportions of EAAs (as determined by the EAAs produced when protein is digested) can also influence the total dietary intake of carbohydrates, perhaps through two different mechanisms.

Our study is the first to examine in detail the nutritional needs for protein/EAA and carbohydrates of the adult worker bumblebee. The bumblebees in our experiments strongly regulated their daily intake of carbohydrate to achieve a minimum of 45 mg sucrose day^−1^. Our previous work has also shown that adult worker honeybees prioritize their intake of carbohydrate over EAAs/protein (Altaye et al., 2010; Paoli et al., 2014a,b; Archer et al., 2014), and that the need for carbohydrate increases when honeybee workers become foragers (Paoli et al., 2014a,b). In our studies with honeybees, we estimated that the IT for newly-emerged honeybees fed the free EAA diets was 1:50 mol/mol. The IT we observed for bumblebees fed the free EAA diets was 1:255 mol/mol – a value that is very similar to our estimate of the IT for honeybee foragers (∼1:250 mol/mol, Paoli et al., 2014a,b). Worker bees have significant demands for carbohydrates to fuel flight (Joos et al., 1997; Suarez et al., 1996; Harrison and Roberts, 2000; Darveau et al., 2014) and also have high resting metabolic rates (Harrison and Roberts, 2000). Their main dietary source of carbohydrates is floral nectar: a solution that contains free AAs but whose composition is largely sucrose, glucose and fructose (Baker and Baker, 1982; Petanidou et al., 2006; Nicolson and Thornburg, 2007). Thus, unlike herbivorous or carnivorous insects, by consuming a nectar-only diet it is possible for foraging bees to selectively consume carbohydrates without being required to eat high concentrations of protein/EAAs at the same time. In this way, they can obtain their carbohydrate needs first, and secondarily consume other substrates (e.g. pollen or glandular secretions from other nest mates) to meet their needs for dietary EAAs.

Honeybees regulate their intake of carbohydrates to maintain haemolymph trehalose titres (Blatt and Roces, 2002a,b). In spite of changes in the quality and quantity of sugar solutions fed to honeybees, the trehalose concentration in haemolymph is tightly regulated to a constant level (Blatt and Roces, 2001, 2002a,b) because trehalose is the main sugar, along with glucose, used to produce glucose-6-phosphate as a substrate for ATP production to fuel flight muscles (Beenakkers et al., 1984). In contrast, we found that in bumblebees, trehalose concentration varied with diet composition, but fructose concentration remained constant. If the maintenance of a storage carbohydrate in haemolymph facilitates flight, we predict that the diet-invariant nature of haemolymph fructose indicates that bumblebees use fructose rather than trehalose to fuel flight. Interestingly, enzymatic studies of bumblebee flight muscles have shown that bumblebees are unique among insects because they rely on fructose-6-phosphate and fructose-1,6-diphosphate as cycling substrates for flight muscles (Staples et al., 2004; Clark et al., 1973; Beenakkers et al., 1984) rather than glucose and glucose-6-phosphate produced from trehalose used by other insects (Beenakkers et al., 1984). Fructose-6-phosphate and fructose-1,6-phosphate can be produced from both glucose and fructose, but the production of fructose-1,6-phosphate – one of the substrates for ATP production in a fructose-6-phosphate/fructose-1,6-phosphate cycle – requires fewer enzymatic steps than it would if trehalose was used as a substrate (Beenakkers et al., 1984; Berg et al., 2012). For this reason, it would be faster and require less ATP for *Bombus* sp. to use fructose than trehalose as a haemolymph storage carbohydrate. Future research on this topic may reveal that fructose plays an important role in the diet of foraging bumblebees for this reason. We also provide the first report we know of that shows sucrose in insect haemolymph, but the significance of this is unknown.

### Diets high in protein lead to eusocial worker mortality

In addition to requiring diets high in carbohydrates to fuel flight, the bumblebees in our study, as well as worker honeybees and ants, also exhibit high rates of mortality when fed diets high in protein or EAAs (Pirk et al., 2010; Paoli et al., 2014a,b; Dussutour and Simpson, 2012) but can survive on a diet of sucrose alone for several days (Paoli et al., 2014a,b). In fact, in addition to having modest demands for dietary protein, bees have significant diversification of genes encoding enzymes necessary for sugar metabolism (Kunieda et al., 2006). Most animals fed diets higher in protein than their actual IT can convert dietary AAs into fuel via gluconeogenesis; we have been unable to find many accounts where diets high in protein kill animals outright, although an abundance of some amino acids, such as methionine, has been associated with toxicity or a reduction in lifespan (Harper et al., 1970; Grandison et al., 2009). These studies, in combination with our data, suggest that the need for diets high in carbohydrates is a general trait of social Hymenoptera workers and could suggest that workers in these lineages have undergone a metabolic trade-off that has perhaps enhanced their ability to use carbohydrates but at the cost of being able to use EAAs efficiently as substrates for energy production.

## MATERIALS AND METHODS

### Experimental animals

Fourteen commercially reared bumblebee (*Bombus terrestris terrestris* Linnaeus 1758) colonies (Koppert Ltd, UK and Syngenta Bioline) were kept in a temperature controlled room or incubators maintained at 28°C and 60% relative humidity at Newcastle University (UK). Prior to the experiment, each colony had access to a liquid food source supplied with the colonies and ∼3 g of honeybee collected pollen was provided daily to each colony. Female worker bees were removed from the colony by opening the flight holes and catching individual bees in plastic vials; bees that emerged from the colony exit were used in the experiments. Bees were briefly cold-anaesthetized on ice until activity was reduced to transfer them into the feeding chambers. Only female bees were used; to identify females, genitals were inspected during cold anaesthesia for the presence of male claspers (Hannan et al., 2012). Workers of all sizes were captured and used in the experiments and care was taken to distribute them randomly across treatments.

### Experimental chambers

Bees were housed individually in a plastic box (16.5×11×6.5 cm) with 20 holes (2 mm) drilled at each end of the lid for ventilation. In three sides of the box, a hole was cut to insert a 2 ml microcentrifuge tube; each tube had four holes (2 mm) drilled in a line in one side of the tube to facilitate feeding by the bees. Two of the tubes were filled with food solution; the remaining tube was filled with deionized water. A piece of absorbent laboratory paper was added to the housing box, covering the base. After being placed in the box, bees were left to acclimatize at room temperature before the feeding solutions were added. Bees were then moved into the 28°C controlled temperature room or incubator and kept in darkness for 7 days, through the course of the experiment. After use in treatments, bees were killed by freezing at −20°C.

### Nutrient balancing experiments and diets

To test how the dietary source of EAAs influenced the intake target of adult worker bumblebees, each bee was presented with a choice of two solutions: a 0.5 mol l^−1^ sucrose solution and another solution that contained 0.5 mol l^−1^ sucrose with protein (sodium caseinate, Sigma-Aldrich, C8654) or the 10 EAAs at equimolar concentrations (Table 5). The AAs used were: methionine, tryptophan, leucine, lysine, valine, arginine, isoleucine, phenylalanine, threonine and histidine (all from Sigma-Aldrich). These AAs are essential for many insect species and were identified as ‘essential’ for honeybees by de Groot (1953). Both of the EAA sources were dissolved in a 0.5 mol l^−1^ sucrose solution made with deionized water. Diets were made to specific protein to carbohydrate ratios (P:C), where the carbohydrate concentration remained constant (0.5 mol l^−1^ sucrose) (Tables 4 and 5). The caseinate solutions were based on weight-to-weight proportions; the EAA solutions were based on the molar ratio of the EAAs-to-sucrose as in Paoli et al. (2014a,b). Our diets did not have the same proportion of EAAs: upon acid hydrolysis (see below), caseinate was digested to a specific proportion of EAA and non-EAA that was dominated by isoleucine, phenylalanine, glutamic acid and tyrosine (Table 4). Furthermore, the most concentrated amino acids were in some cases three or four orders of magnitude higher than the least concentrated amino acids. In contrast, our EAA diet was nearly equimolar with a similar proportion w/w.

**Table 5.**
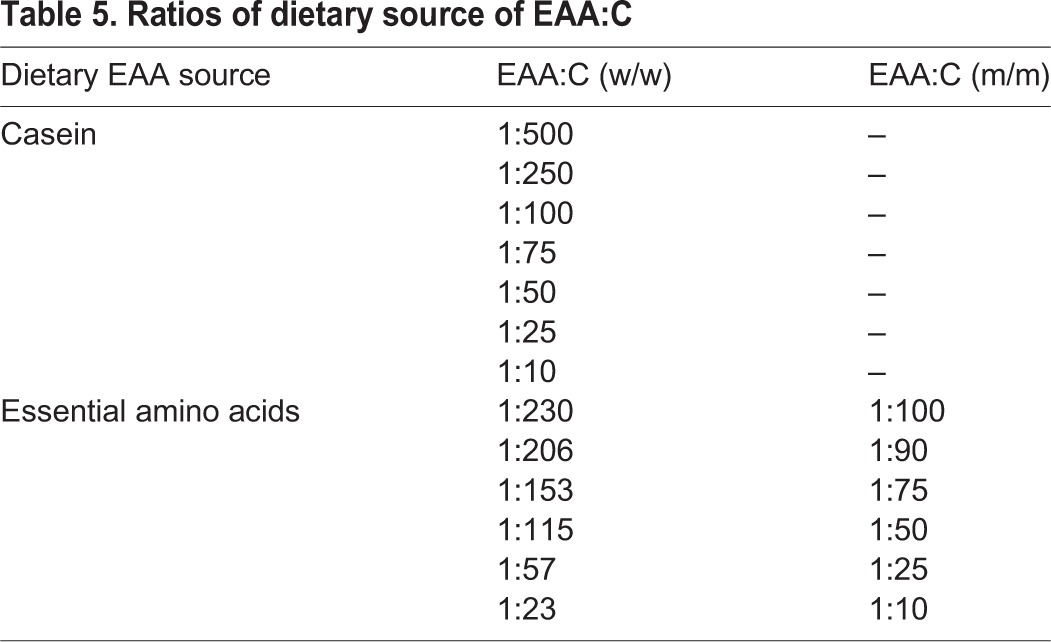
**Ratios of dietary source of EAA:C**

Diet tubes were weighed and replaced every 24 h. To adjust for evaporation, evaporation rates for each solution were measured in boxes containing the solutions (without bees). The average value for each solution was subtracted from the final weights for the consumption of each diet solution. Values for the amount of carbohydrate or protein and EAAs consumed were determined by dividing the weight of the consumed solution by its density (1.06) to obtain the volume. The amount of each solute in the solution was then obtained for the volume of solution consumed; this amount was combined to give a single value for consumption of protein and carbohydrate for each day. Total consumption was a measure of the total amount eaten over the 7 day period.

### Effect of diet on haemolymph composition

We measured haemolymph sugars and AAs with the aim of identifying how diets of caseinate or EAAs influenced nutritional state and hence nutrient balancing (Simpson and Raubenheimer, 1993). To do this, we restricted individual bees for 3 days to one of the following diets: sucrose only, low caseinate (1:140 w/w), high caseinate (1:20 w/w), low EAAs (1:600 mol/mol), or high EAAs (1:30 mol/mol) using the protocol described above. After 3 days, haemolymph was collected from each bee. Bees were cold-anaesthetized, and a hypodermic needle was used to cut an incision in the back of the head posterior to the ocelli. Haemolymph from individual bees was collected using 10 µl capillary tubes and expelled into a 0.2 ml microcentrifuge tube with an equal volume of 0.1 mol l^−1^ perchloric acid to haemolymph. Each sample represented haemolymph from one bee. The average volume of haemolymph collected from an individual was 6.16 µl (the volume of haemolymph did not differ between treatment groups). Samples were kept frozen at −20°C until HPLC analysis. For HPLC analysis, 4 µl of haemolymph-perchloric acid mixture from each bee was diluted to 1:30 with HPLC gradient grade H_2_O. Each sample was passed through a 0.45 μm syringe-tip filter (Whatman Puradisc 4, nylon, 4 mm) prior to analysis.

In each sample, we used HPLC to measure glucose, trehalose, fructose and sucrose and a suite of EAAs and non-EAAs. Sugars were quantified using a Dionex DX500 HPLC system with an ED40 electrochemical detection unit. The mobile phase was 100 mmol l^−1^ NaOH. A separate aliquot of the original haemolymph-perchloric acid sample was diluted to 1:200 with distilled, deionized water. Twenty microlitres of this sample was injected on to a Carbopac PA-100 column (Dionex, Sunnyvale, California, USA). Sugars were eluted isocratically with 100 mmol l^−1^ NaOH with a flow rate of 1 ml min^−1^. Elution profiles were analysed with Chromeleon software (Thermo Fisher Scientific).

We quantified 21 AAs in the samples using a Dionex Ultimate 3000 RS system fitted with a 150×2.1 mm Accucore RP-MS (Thermo Scientific) column. Before being injected onto the column, 10 µl of diluted sample was pre-treated for 1 min with 15 µl of 7.5 mmol l^−1^ o-phthaldialdehyde (OPA) and 225 mmol l^−1^ 3-mercaptopropionic acid (MPA) in 0.1 mol l^−1^ sodium borate (Na_2_B_4_O_7_·10H_2_O, pH 10.2), then with 10 µl of 96.6 mmol l^−1^ 9-fluroenylmethoxycarbonyl chloride (FMOC) in 1 mol l^−1^ acetonitrile for 1 min, followed by the addition of 6 µl of 1 mol l^−1^ acetic acid. A final volume of 30 µl of the treated sample was then injected into the HPLC system. Elution solvents used were: A, acetonitrile/methanol/water (45/45/45 v/v/v) and B, 10 mmol l^−1^ Na_2_HPO_4_, 10 mmol l^−1^ Na_2_B_4_O_7_·10H_2_O, 0.5 mmol l^−1^ sodium azide (NaN_3_), adjusted to pH 7.8 with concentrated HCl. Elution of the column occurred at a constant flow rate of 500 µl min^−1^ with a linear gradient of 3 to 100% (v/v) eluent A and 97 to 0% eluent B. Amino acid derivatives were fluorometrically detected (Ultimate 3000 RS Fluorescence Detector, Dionex, Thermo Fisher Scientific) and elution profiles were analysed using Chromeleon software (Thermo Fisher Scientific).

### Amino acid composition of sodium caseinate

To identify the AAs produced by the digestion of caseinate, we digested sodium caseinate in HCl. Sodium caseinate (1.7 mg) was first washed in 200 µl of methanol to extract free AAs. The samples were vortexed for 1 min and then left for 10 min and vortexed a second time for 1 min (Cook et al*.*, 2003). Each sample was centrifuged for 30 min at 134,000 r.p.m. The supernatant was removed and placed in a new microcentrifuge tube. The remaining pellet and the supernatant sample were dried down in a heat block at 70°C. Dry samples from the methanol extract were then recovered in 200 µl HPLC gradient grade water and vortexed for 1 min. To the dried caseinate pellet, we added 170 µl of 6 mol l^−1^ hydrochloric acid (HCl) and the sample was briefly vortexed. Sealed tubes were placed in plastic microcentrifuge tube boxes, sealed, and placed in a domestic 900 W (2450 MHz) microwave oven inside of a fume hood. A Pyrex beaker containing 800 ml of cold tap water was also placed in the microwave oven to absorb excess radiation (Zhong et al., 2005). Samples were irradiated for 15 min on full power and then left to cool. Cooled samples were then moved to a heat block within a fume hood, unsealed and heated at 70°C to evaporate the acid. Once dry, 200 µl of deionized UHPLC gradient grade water was added to each sample. Both free AA (supernatant) and hydrolysed protein-bound AA samples (digested pellet) were centrifuged for 1 min and filtered through 0.45 µm syringe-tip filters (Whatman Puradisc 4, nylon, 4 mm). Ten microlitres of each filtered sample was analysed using the HPLC method for AA analysis above.

### Statistical analyses

Analyses were carried out using IBM SPSS v19. The amount of food consumed (mg) was analysed using multivariate analysis of variance (MANOVA) and the volume analysed using two-way analysis of variance (ANOVA). Both analyses included colony and bee size as covariates. Šidák's *post hoc* tests were used for multiple comparisons. Data were natural log transformed prior to analysis. (Note that we used bee weight as a proxy for bee size based on a factor analysis that identified that bee weight had the strongest correlation with four other measured parameters: abdomen and thorax width, head length, total bee length.) The intake targets were determined by the *post hoc* comparisons of the amount of protein/EAAs and carbohydrates eaten on each dietary treatment; diet treatments that were not significantly different in both were averaged to determine the intake target P:C or EAA:C ratio. Survival data were analysed using a Cox regression (Coxreg) analysis with diet treatment as a covariate; comparisons between groups were evaluated using the ‘indicator’ contrasts. The hazard ratio (HR) was calculated for each comparison against the indicator group, which was always the most dilute EAA:C or P:C treatment. Generalized estimating equations (GEEs) were used to test for differences in haemolymph sugars and total EAA and non-EAA. A canonical discriminant analysis (CDA) was used to identify differences in the treatments in the amount and proportion of specific AAs in haemolymph.

## Supplementary Material

Supplementary Material
