# Supplementary material for: Nutrient balancing of the adult worker bumblebee (Bombus terrestris) depends on the dietary source of essential amino acids
Source: J Exp Biol. 2015 Mar;218(5):793–802. doi: 10.1242/jeb.114249 (PMC4376193; doi:10.1242/jeb.114249)
Supplement: Supplementary Material [file supp_218_5_793__index.html]

Nutrient balancing of the adult worker bumblebee (Bombus terrestris) depends on the dietary source of essential amino acids — Supplementary Material 

# Nutrient balancing of the adult worker bumblebee (*Bombus terrestris*) depends on the dietary source of essential amino acids

## JEB114249 Supplementary Material

**Files in this Data Supplement:**

- **Supplementary Material**
