# Supplementary material for: Nutrient balancing of the adult worker bumblebee (Bombus terrestris) depends on the dietary source of essential amino acids
Source: J Exp Biol. 2015 Mar;218(5):793–802. doi: 10.1242/jeb.114249 (PMC4376193; doi:10.1242/jeb.114249)

Fig. S1. Regulation of carbohydrates. To identify the concentration used for the paired diet solutions with protein and amino acids, we tested the carbohydrate regulation of individual worker bumblebees. Bees were provided for 6 days with one of the pairs of the following sucrose solutions: 1) 0.5M with 0.1M; 2) 0.5M with 0.01M; 3) 0.25M with 0.1M; or 4) 0.25M with 0.01M. (A) When given the choice between two sucrose solutions, bumblebees always chose to feed on the higher concentration solution . (B) We also tested consumption for two different pairs of diets: Bumblebees ate nearly twice as much of 0.25M sucrose as the 0.5M sucrose solution. In fact, on average, bumblebees maintained an intake target of approximately 30mg of sucrose per day. This, in combination with the data presented in Fig.1A IT=1:140 (~70mg per day) and Fig. 1B IT=1:570 (~30-40mg per day) strongly suggests that the intake target for an adult worker bumblebee is between 30-70mg of carbohydrate per day. Intake was balanced so that bumblebees consumed the same amount of carbohydrate irrespective of the concentration of the solution provided (repeated measures ANOVA F=0.005, P = 0.947)

Figure S1

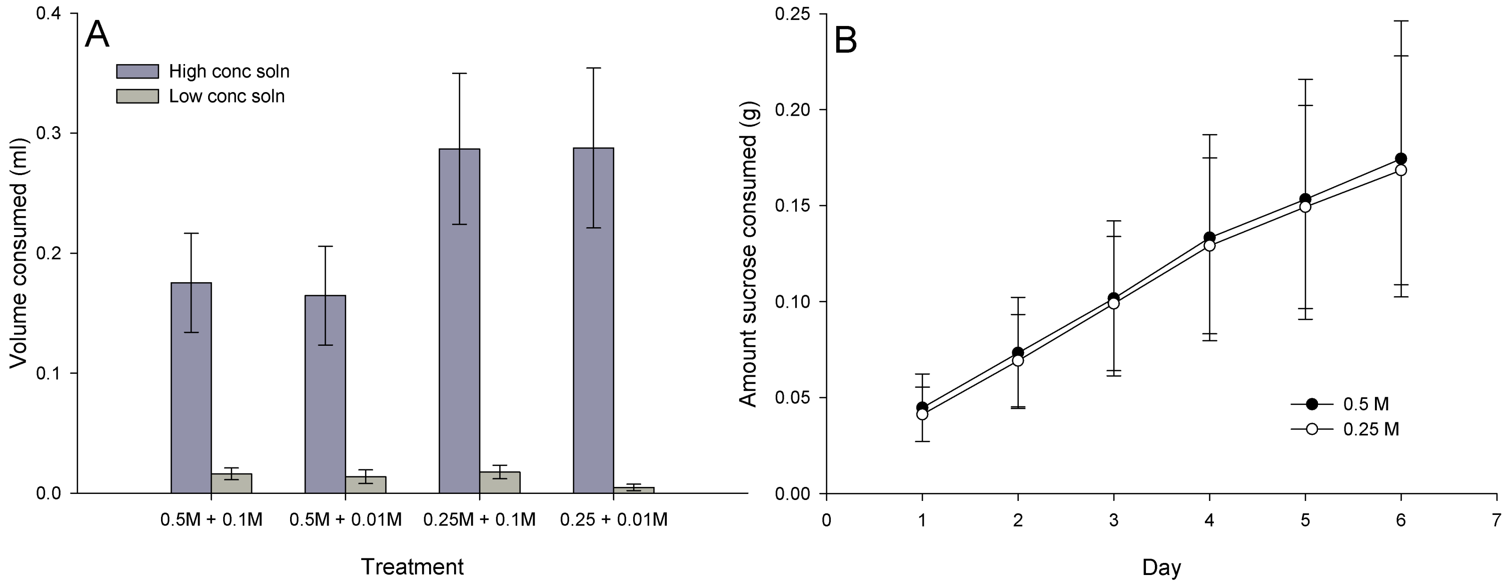

Supplement: Supplementary Material [file supp_218.5.793_JEB114249.pdf]
